# Chronic Eosinophilic Pneumonia: Unraveling Recurrent Chest Infections in a Female Patient With a Family History of Atopy

**DOI:** 10.7759/cureus.49743

**Published:** 2023-11-30

**Authors:** Zaheer Aslam, Omer Chowdhary, Yuhui Zhou, Anjana Razik, Mostafa Negmeldin

**Affiliations:** 1 Respiratory Medicine, Bedfordshire Hospitals NHS Foundation Trust, Bedford, GBR; 2 General/Internal Medicine, Luton and Dunstable University Hospital, NHS Foundation Trust, Luton, GBR; 3 Internal Medicine, Cambridge University Hospitals, Cambridge, GBR; 4 General/Internal Medicine, Bedfordshire Hospitals NHS Foundation Trust, Bedford, GBR; 5 Pulmonology, Bedfordshire Hospitals NHS Foundation Trust, Bedford, GBR

**Keywords:** atopy, prednisolone, bronchoscopy, eosinophilia, eosinophilic pneumonia

## Abstract

Eosinophilic pneumonia is a rare condition characterized by the infiltration of eosinophils in the lungs. We present a case of eosinophilic pneumonia in a 51-year-old British Caucasian female with a history of lichen sclerosus, deranged liver function tests, and a family history of atopy. The patient presented with fever, shortness of breath, lethargy, dry cough, and weight loss over a three-month period. Initial treatment with antibiotics did not yield improvement, and further investigations revealed marked eosinophilia on blood count. Bronchoscopy and biopsies confirmed the diagnosis of eosinophilic pneumonia, and the patient responded well to a tapering dose of prednisolone. This study highlights the importance of considering eosinophilic pneumonia in patients with unexplained respiratory symptoms and eosinophilia and emphasizes the role of bronchoscopy in establishing a definitive diagnosis.

## Introduction

Eosinophilic pneumonias are a group of rare heterogeneous conditions characterized by eosinophilic infiltration of lung tissue, accounting for 2.5% of interstitial lung disease [1]. They can be classified as acute or chronic and can be further categorized based on etiologies which include parasitic infections, medications, autoimmune and inflammatory conditions, and termed idiopathic if the etiology is unknown. Eosinophilic pneumonias can present with a wide range of symptoms and are often associated with peripheral eosinophilia. We present a case of idiopathic chronic eosinophilic pneumonia with peripheral eosinophilia in a middle-aged female patient with a background of atopy, highlighting the importance of considering a thorough history and the diagnostic challenges of this condition.

## Case presentation

A White female in her 50s had a fever and cough for one month before presenting to her general practitioner (GP). Initial evaluation by the GP led to two courses of doxycycline for lower respiratory tract infection without improvement. On her hospital admission two weeks later, her main symptoms were fever, shortness of breath, lethargy, dry cough, and weight loss. Her past medical history was notable for lichen sclerosus diagnosed in her late teenage years, for which she was treated with topical steroids for many years including a course of oral steroids two months before the onset of her respiratory symptoms. She also struggled with muscle aches over months and was previously seen at her GP clinic for fibromyalgia. She suffered from joint pain after exercise and 20 minutes of early morning stiffness. She did not have sicca symptoms but had Raynaud’s phenomenon for many years. Her medication history was otherwise significant for hormone replacement therapy for menopause. In her social history, she worked as a hairdresser and in a variety of administrative roles since leaving school. She had never smoked and had no other known chemical/mold/animal exposure apart from hair dye while working as a hairdresser and a pet dog for the last 12 years. She lived in Abu Dhabi 13 years ago for two years, during which she mainly stayed at home as a housewife. There is a significant history of atopy in the family; her son and mother both suffer from allergies and asthma. Her father has Addison’s disease. On admission, general examination revealed no skin lesions, temperature was 36.7 degrees, oxygen saturation was 96% on room air, respiratory rate was 18 per minute, and blood pressure was 126/85 mm of Hg. She was tachycardic with a heart rate of 104 beats per minute. Auscultation of the lungs demonstrated bilateral upper lobe crackles. The rest of the examination was unremarkable. She, however, had low oxygen saturation while on the ward and required oxygen at the rate of 1 liter/minute through a nasal cannula to maintain normal saturations. She was successfully weaned off oxygen after starting steroid treatment.

Investigations

**Figure 1:**
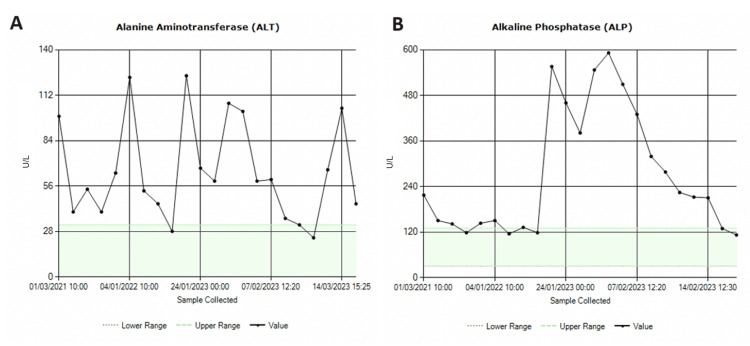
Trends in liver function test. Images A and B show ALP and ALT levels, respectively. The derangement in liver function tests could be due to a course of antibiotics previously taken by the patient and unrelated to the chronic eosinophilic pneumonia. ALP: alkaline phosphatase; ALT: alanine transaminase

One factor that led to her initial admission to the hospital was deranged laboratory investigations. On admission, her peripheral eosinophil count was 2.07 × 10^9^/L. Her liver function tests (LFTs) were deranged with the trend shown in Figures 1A, 1B, which outlines levels from the time of her initial hospital admission. The hepatitis screen was negative.

**Figure 2:**
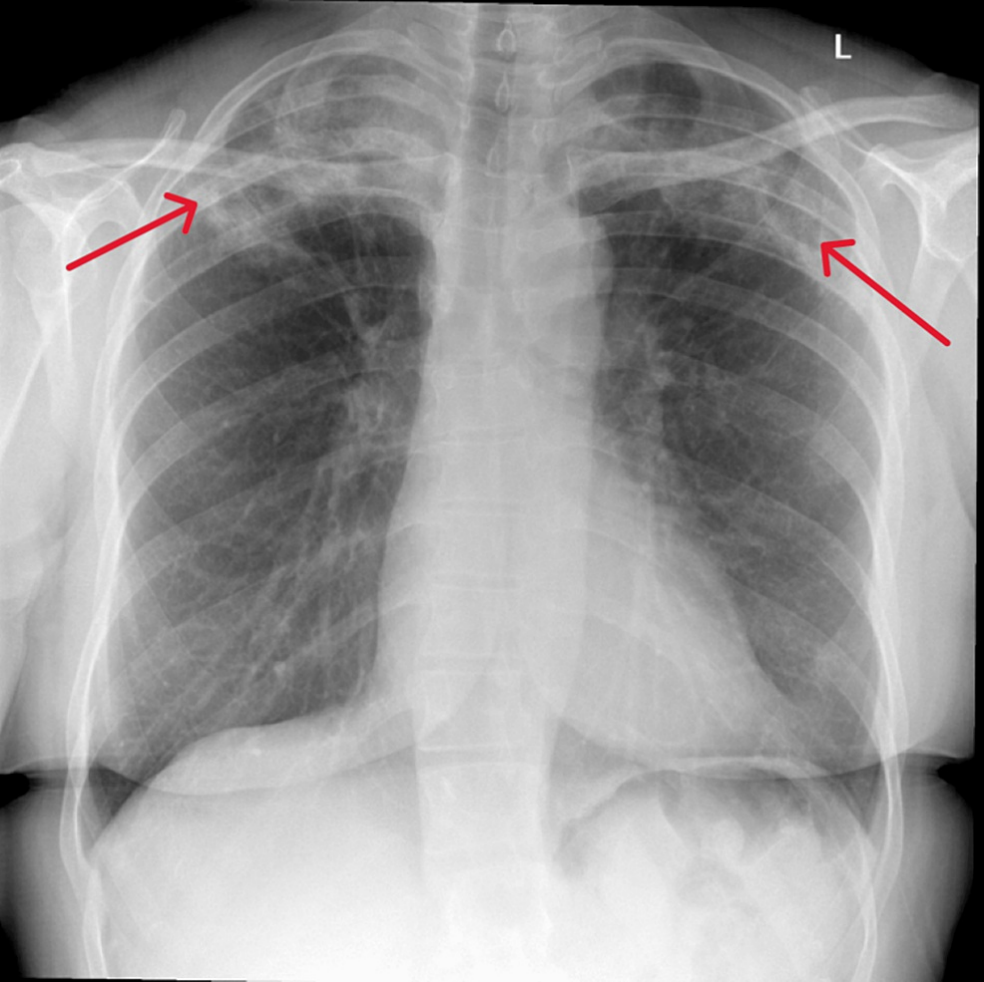
Chest X-ray taken on initial presentation. Arrows point to bilateral upper lobe opacification.

A CT scan of the abdomen showed no significant abdominal or pelvic pathology. Angiotensin-converting enzyme (ACE) levels were within normal limits. The basic autoimmune panel was positive for a weak Ro Antibody on myositis line blot, however, was otherwise negative, notably for antinuclear antibody (ANA)-HEp-2. On initial presentation to her GP, a chest X-ray was taken showing bilateral upper lobe opacification (Figure 2).

**Figure 3:**
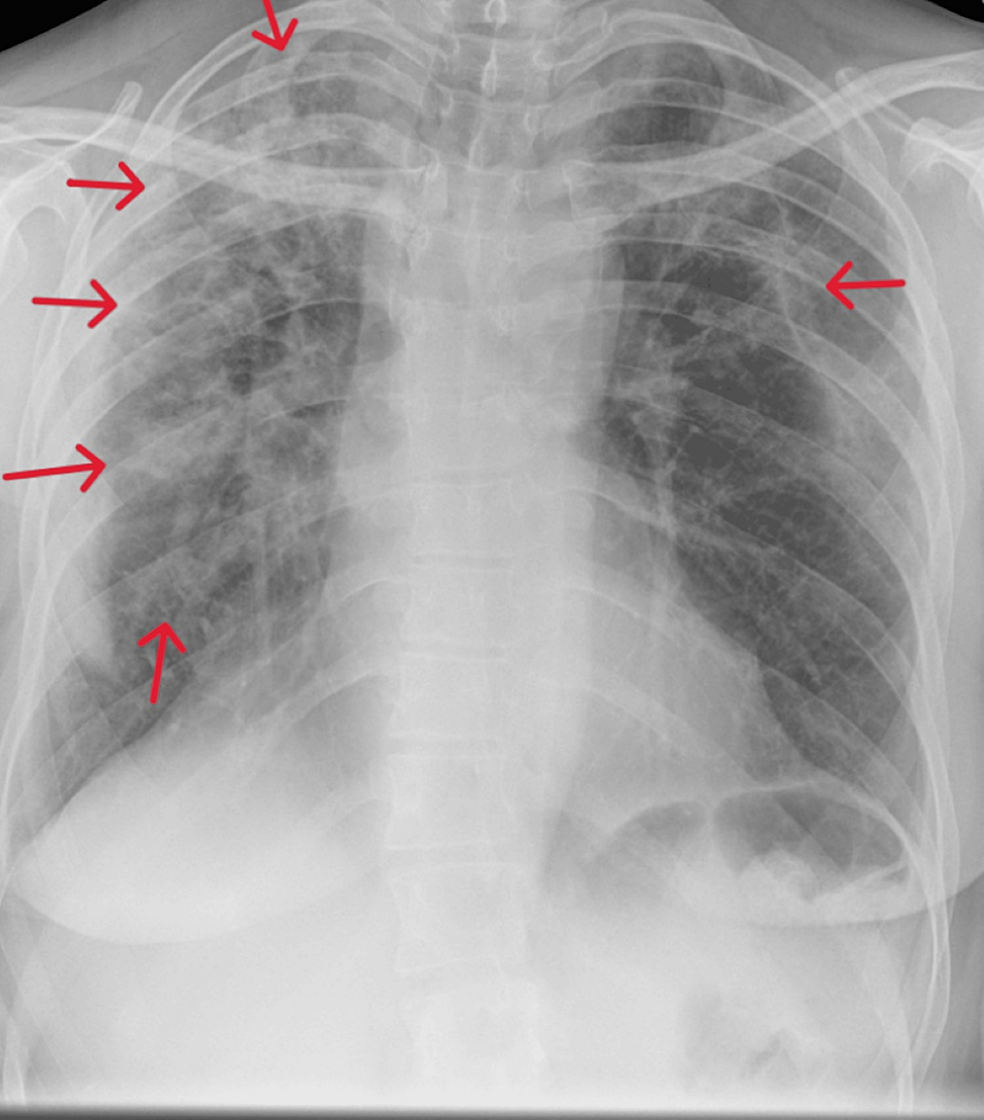
Repeat chest X-ray was conducted two weeks after the patient's admission. Arrows indicate worsened bilateral opacification compared to Figure 2.

**Figure 4:**
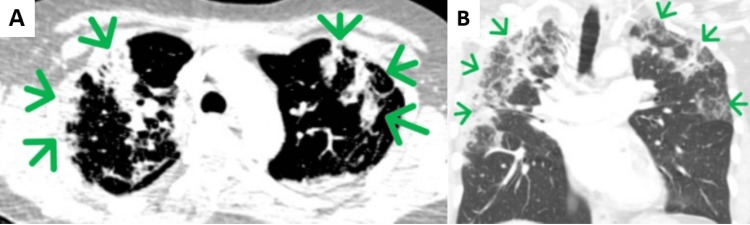
High-resolution CT scan of the chest. Arrows show areas of peripheral consolidation in both axial (A) and coronal (B) views.

On her admission, a repeat chest X-ray taken two weeks later showed worsened bilateral upper zone opacification (Figure 3). High-resolution chest CT as shown in Figures 4A, 4B showed a peripheral consolidative pattern in keeping with eosinophilic pneumonia. She underwent an inpatient bronchoscopy procedure due to her non-productive cough and unclear cause of her radiological findings. Bronchoalveolar lavage (BAL) was performed from the apical segment of the right upper lobe and two specimens (A and B) were taken which contained pulmonary macrophages, lymphocytes, polymorphs, and numerous eosinophils (84% in specimen A and 74% in specimen B). BAL fluid culture did not grow any bacteria or fungi. Cultures on Löwenstein-Jensen (LJ) medium showed no acid-fast bacilli (AFB) growth. Blood IgE levels were normal. Specific IgE for *Aspergillus fumigatus* and antinuclear cytoplasmic antibodies (ANCA) test was negative. Lung function tests were not performed during admission as the patient was unwell and therefore unable to perform a spirometry of acceptable quality.

Differential diagnosis

Our main differentials were respiratory or systemic conditions that present with peripheral shadows and lung eosinophilia. Potential etiologies considered include drug-induced reactions involving substances like antimalarials and sulphonamides which were excluded, parasitic and fungal infections that contribute to the observed symptoms, eosinophilic granulomatosis with polyangiitis, and neoplasms and idiopathic acute/chronic eosinophilic pneumonia. Hypereosinophilic syndrome and allergic bronchopulmonary aspergillosis were also on the list. The patient was not on any medication that could have potentially caused a drug reaction. Stool examination for ova, cysts, and parasites and serology tests for parasites like Schistosoma and Strongyloides were negative. BAL mycology and fluid culture for fungi were also negative making fungal and parasitic infections an unlikely cause of the presenting symptoms. Eosinophilic granulomatosis with polyangiitis had a low probability as the ANCA test was negative. Allergic bronchopulmonary aspergillosis was ruled out as specific IgE for *Aspergillus fumigatus* was negative. Persistent eosinophilic count for more than 12 weeks pointed towards a chronic etiology rather than an acute one. Idiopathic form of eosinophilic pneumonia became more likely as extrinsic etiologies were excluded with the help of a thorough history and specific investigations.

Treatment

**Figure 5:**
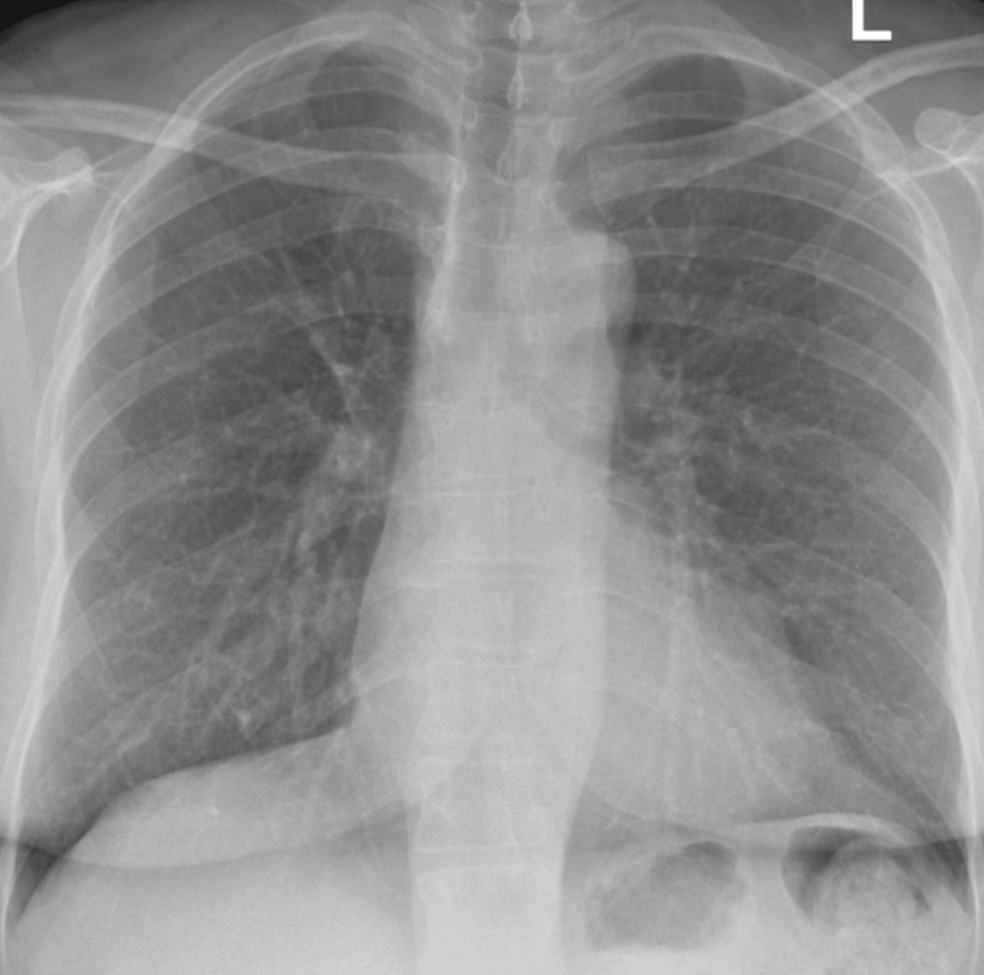
Chest X-ray taken during the first outpatient follow-up visit one month after discharge. The image shows significant improvement when compared to the chest X-ray from four weeks ago.

**Figure 6:**
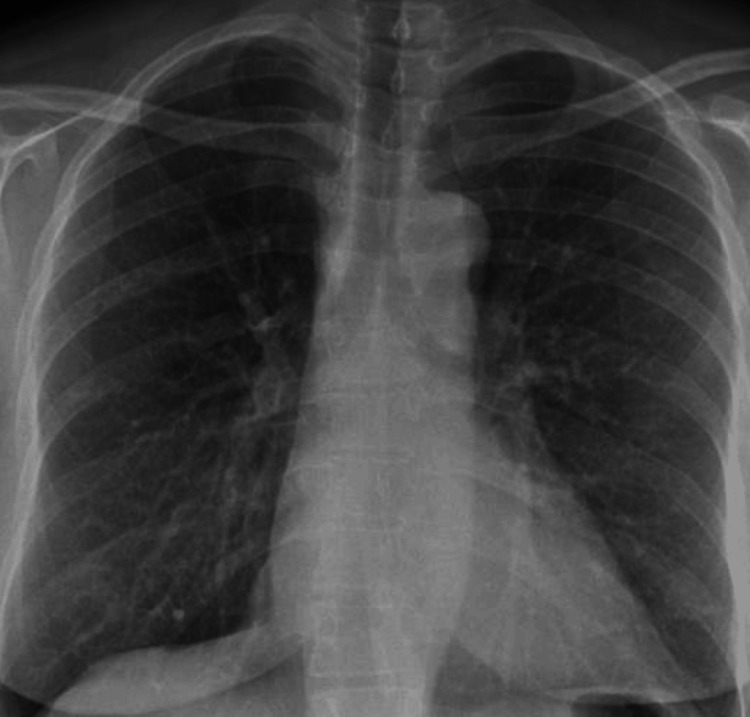
Chest X-ray taken six months after discharge from the hospital. The image shows complete resolution of consolidation as a result of excellent response to steroid therapy.

The patient was initially discharged from the first admission while waiting for BAL results and outpatient clinic follow-up, however, was re-admitted one week later due to worsening symptoms. Given the main suspicion of eosinophilic pneumonia after BAL, the patient was initiated on a trial of tapering prednisolone at 60 mg and dropping 5 mg per week along with a proton pump inhibitor cover. Chest X-ray taken during the first follow-up visit was fairly normal with significant improvement when compared to the chest X-ray from four weeks ago (Figure 5). Chest X-ray six months after discharge showed complete resolution of consolidation seen on previous chest X-rays (Figure 6).

Outcome and follow-up

**Figure 7:**
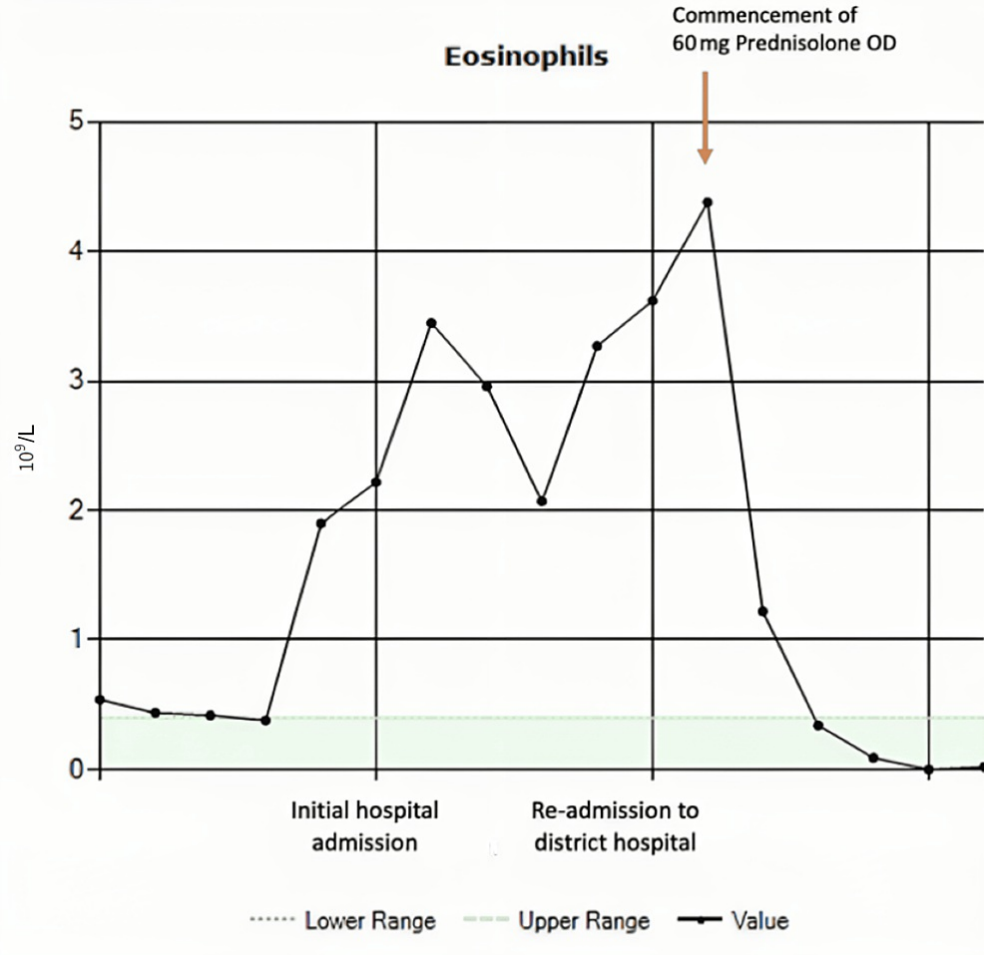
Trend in peripheral eosinophil count. There was a rapid decline in eosinophil count from the point of starting steroid treatment indicated by the red arrow.

Figure 7 shows the trend in peripheral eosinophil count highlighting the decline in levels from the point of starting steroid treatment. She was subsequently followed up in a tertiary center under private healthcare insurance to monitor her symptoms, lung function, and laboratory results. Two months after discharge, the patient’s exercise tolerance improved significantly. She was able to walk 5 miles which was not possible on the initial presentation.

**Figure 8:**
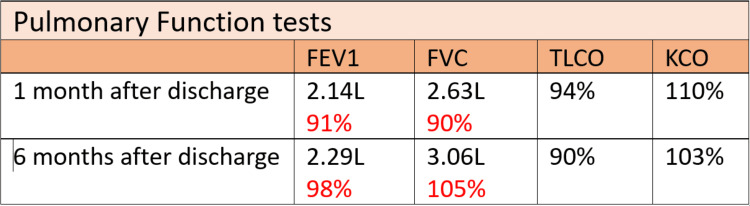
Pulmonary function tests were done during regular outpatient follow-up visits after the admission. The figure shows the results of the tests done one month and six months after discharge from the hospital and that the pulmonary function tests are normal. There is an improvement in forced vital capacity (FVC) by 400 mL in repeat test. Diffusion capacity is also within the normal range. FEV1: forced expiratory volume in 1 second; TLCO: transfer capacity of the lung; KCO: carbon monoxide transfer coefficient

Pulmonary function tests done during the first follow-up after a month were normal (Figure 8). Forced vital capacity (FVC) was 2.63 L which was 90% of predicted. Lung function tests repeated after four months showed a further increase, with her FVC increasing by 400 mL to 3.06 L which was 105% of predicted. Diffusion capacity remained within the normal range. Given her good response, she was advised to taper down to a maintenance dose of 10 mg of prednisolone to keep her symptoms under control. Nine months after her discharge she continued to be on the same maintenance dose with no relapses. Her GP plans to taper her prednisolone in 1 mg increments with continuous monitoring of blood count and inflammatory markers. LFTs remained deranged with an alanine transaminase (ALT) of 127 U/L and gamma-glutamyl transferase (GGT) of 177 U/L when tested two months after discharge but normalized when repeated four months later. As the investigations, including a magnetic resonance cholangiopancreatography (MRCP), done as an outpatient procedure were inconclusive, the plan is to monitor with regular liver function tests and perform a liver biopsy if there is a further surge in the levels.

## Discussion

There is a 2:1 female predominance and a mean age of 45 years at diagnosis for eosinophilic pneumonia, which is fairly consistent with the case presented here [2]. Given the patient’s eosinophilic count was elevated over 12 months, this was more in keeping with chronic eosinophilic pneumonia (CEP). Many extrinsic etiologies were excluded from a thorough history and laboratory investigations in the case presented, making this most likely a case of idiopathic CEP. The most notable positive finding was the patient’s significant family atopic history. The link between eosinophilic pneumonia and allergic diseases, such as asthma, atopic dermatitis, and allergic rhinitis, has been shown previously [3]. For example, some, but not all patients diagnosed with eosinophilic pneumonia have a medical background of asthma, while some develop asthma after a diagnosis of eosinophilic pneumonia [4]. The patient we presented may benefit from close monitoring of the development of asthmatic symptoms. To our knowledge, no current case reports have described an association between eosinophilic pneumonia and lichen sclerosus. However, eosinophilic infiltration, which is a hallmark of eosinophilic pneumonia, has been described in lichen sclerosus and linked to lichen sclerosus-associated squamous cell carcinoma [5].

Part of the diagnostic challenge lies with the lack of specific symptoms of CEP. Non-respiratory, general systemic symptoms, such as weight loss and lethargy, are also commonly described. A high degree of clinical suspicion is required for a diagnosis of CEP. Deranged LFTs are commonly reported in cases of liver involvement in eosinophilic pneumonia secondary to parasitic/helminth infection. Parasitic/fungal screen tests were negative and travel/occupational history was low risk in this case. To our knowledge, deranged LFTs have not been previously associated with idiopathic CEP. This could be unrelated to CEP or linked to the use of previous courses of antibiotics. Hypercalcemia has rarely been reported in CEP, however, given its resolution and raised parathyroid hormone (PTH), this is likely a separate pathology. Bronchoscopy results are strong evidence if show eosinophilia. The patient showed excellent response to oral prednisolone therapy, which is consistent with previous case reports [6]. Studies have shown that long-term oral corticosteroid therapy is necessary in up to half of patients because of the high rates of relapse [4]. This raises the need to monitor patients for adverse effects of chronic steroid therapy including high blood glucose, weight gain, and gastrointestinal symptoms. Essentially, a thorough history and laboratory investigation panel are required for a diagnosis of eosinophilic pneumonia. Treatment response is generally favorable, however, the adverse long-term steroid therapy must be closely monitored and weighed up against the benefits.

## Conclusions

Eosinophilic pneumonia commonly presents with non-specific symptoms and signs, resulting in diagnostic challenges. There is a link between eosinophilic pneumonia and atopy and it may also be associated with inflammatory conditions like lichen sclerosus. Response to steroids in eosinophilic pneumonia is generally favorable.
